# Serum Starvation Affects the Transcriptomic and Proliferative Response to ACTH in Primary Cultures of Rat Adrenocortical Cells

**DOI:** 10.3390/cells14231844

**Published:** 2025-11-22

**Authors:** Małgorzata Blatkiewicz, Emilia Cicha, Marta Szyszka, Karol Jopek, Marianna Tyczewska, Izabela Pieścikowska, Ludwik K. Malendowicz, Marcin Rucinski

**Affiliations:** 1 Department of Histology and Embryology, Poznan University of Medical Sciences, 60-781 Poznan, Poland; mblatkiewicz@ump.edu.pl (M.B.); mszyszka@ump.edu.pl (M.S.); kjopek@ump.edu.pl (K.J.); maritycz@ump.edu.pl (M.T.); marcinruc@ump.edu.pl (M.R.); 2 Animal Facility, Poznan University of Medical Sciences, Rokietnicka 8, 60-806 Poznan, Poland; emiliacicha@ump.edu.pl; 3 Department of Anatomy and Histology, University of Zielona Gora, Licealna Street 9, 65-417 Zielona Gora, Poland; ipiescikowska@ump.edu.pl; 4 Department of Bioinformatics and Computational Biology, Poznan University of Medical Sciences, Swiecickiego 6, 60-781 Poznan, Poland

**Keywords:** ACTH, cell proliferation, steroidogenesis, serum starvation, transcriptomic profiling

## Abstract

The adrenocorticotropic hormone (ACTH) is a key regulator of adrenal cortex function, promoting glucocorticoid synthesis and modulating cell proliferation. However, the role of extracellular steroid availability in shaping ACTH responses is still not fully defined. In this study, the functional and transcriptomic effects of ACTH were investigated in primary rat adrenocortical cells cultured under standard conditions and under simulating serum starvation (charcoal-stripped serum). The cells were treated with ACTH (10 nM), and proliferation was monitored using xCELLigence RTCA, while corticosterone secretion was assessed via ELISA. The RNA extracted from these samples was then utilised for the purpose of microarray-based gene expression profiling. The present study revealed that charcoal-stripped serum markedly improved ACTH-induced corticosterone output, suggesting that the absence of endogenous steroids sensitises cells to ACTH stimulation possibly by removing negative feedback constraints. This enhanced steroidogenic response was accompanied by a significant suppression of proliferation, confirming that the stimulation of specialised functions (such as steroid secretion) reduces proliferative capacity of adrenocortical cells. Transcriptomic data revealed that the steroids stimulating effect on corticosterone output was mainly mediated via steroid biosynthetic and lipid metabolic processes while inhibitory effect on proliferation rate was mediated mainly by cell adhesion molecules. These results suggest that, in primary culture of rat adrenocortical cells, the stimulatory effect of ACTH on their specialised function (corticosteroid secretion) simultaneously reduces their basal function, which is their proliferation process. Changes in this type are also observed in cells cultured in steroid-depleted conditions.

## 1. Introduction

ACTH was first isolated in 1943 and chemically synthesised in the 1970s. It plays a central role in the regulation of homeostasis and the physiological response to stress by stimulating corticosteroid synthesis in the adrenal cortex. It functions as a pivotal effector of the hypothalamic–pituitary–adrenal (HPA) axis, orchestrating neuroendocrine responses to metabolic, immune, and psychological stressors [1,2,3,4]. ACTH is synthesised and secreted by corticotroph cells of the anterior pituitary and acts predominantly on the zona fasciculata and reticularis of the adrenal cortex, where it binds with high specificity to melanocortin 2 receptors (MC2R), initiating a cAMP–PKA–dependent signalling cascade [5,6]. This results in the upregulation of key steroidogenic genes, including *StAR*, *CYP11B1*, and *HSD3B2* [7,8]. It is important to note that ACTH exerts its influence not only on the synthesis of glucocorticoids but also on functions such as adrenal growth, vascular tone, and cellular plasticity [9,10].

The adrenal glands, paired endocrine organs located on the kidneys, are structurally divided into three concentric cortical zones: zona glomerulosa (aldosterone), zona fasciculata (cortisol in human or corticosterone in rodents), and zona reticularis (androgens) [11]. While the synthesis of aldosterone is primarily governed by angiotensin II and potassium, the production of glucocorticoids is subject to tight regulation by ACTH via *MC2R*, whose expression is most prominent in the zona fasciculata [12]. The synthesis of steroid hormones by the adrenal cortex has been demonstrated to regulate a broad range of physiological functions, including water-electrolyte balance, energy metabolism, immune modulation, and behavioural stress responses [13,14]. In addition to systemic ACTH signalling, recent studies highlight the importance of intra-adrenal regulatory mechanisms, including autocrine and paracrine input from chromaffin cells, immune cells, adipocytes, and sympathetic innervation. Furthermore, circadian clock systems, both central and intra-adrenal, contribute to the temporal regulation of ACTH secretion and responsiveness, thereby aligning steroid production with daily physiological rhythms [15,16].

Although the role of ACTH in steroidogenesis is well established, its effects on proliferation, differentiation, and functional reprogramming of adrenocortical cells remain incompletely understood, especially under conditions of altered hormonal feedback. In vitro studies have demonstrated that steroid depletion, for instance through the use of charcoal-stripped serum, can render adrenal cells more susceptible to ACTH stimulation [17,18]. This effect is presumably attributable to the removal of glucocorticoid-mediated negative feedback. In such conditions, cells may exhibit exaggerated steroidogenic responses and altered proliferation dynamics [17,19].

The aim of the performed experiments was to investigate the mechanisms/intracellular signalling pathways through which, in primary cultures of rat adrenocortical cells, ACTH regulates steroidogenesis and proliferation and this under conditions of different steroid concentrations in the incubation medium (modulated by charcoal-stripped serum). To explore the molecular mechanisms underlying ACTH responsiveness, a multi-disciplinary approach was employed, incorporating functional, biochemical and transcriptomic analyses. The hypothesis was formulated that steroid depletion would amplify the steroidogenic response to ACTH while simultaneously suppressing cell proliferation, reflecting a functional “trade-off” between hormone production and cell cycle progression.

## 2. Materials and Methods

### 2.1. Primary Rat Adrenocortical Cell Culture

All experimental procedures involving animals were approved by the Local Ethics Committee for Animal Experimentation (approval number: 27/2025; date of approval: 25 April 2025). Male Wistar rats (aged 10 weeks) were maintained under controlled environmental conditions (temperature 22–24 °C, a 12 h light/dark cycle) ad libitum access to food and water. Prior to adrenal gland collection, animals were euthanized by rapid decapitation. The primary cultures were prepared by carefully dissecting and enzymatically digesting the adrenal glands, followed by filtration and centrifugation. To minimise contamination from the capsule and adipose tissue in this study, the glands were carefully dissected before enzymatic digestion by (i) removing fat and connective tissue under a stereomicroscope; (ii) discarding the medullary tissue and visible capsular fat; and (iii) using a 70 µm filter to remove larger fragments. Adrenal glands from 20 rats were promptly removed, rinsed in ice-cold phosphate-buffered saline (PBS, pH 7.4; Sigma-Aldrich, St. Louis, MO, USA), and transferred into Petri dishes containing phenol red-free Dulbecco’s Modified Eagle’s Medium/F12 (DMEM/F12; Sigma-Aldrich, Cat. No. D6434, St. Louis, MO, USA). Under sterile conditions in a laminar flow hood, excess fat and connective tissue were meticulously dissected from each gland. Glands were then minced into approximately 1 mm^3^ fragments and enzymatically digested in DMEM/F12 containing collagenase type I (1 mg/mL, Sigma-Aldrich, Cat. No. SCR103, St. Louis, MO, USA) at 37 °C for 30 min in a shaking water bath. Following digestion, tissue disintegration was facilitated by gentle pipetting using a sterile glass Pasteur pipette (Brand, Wertheim, Germany). The resulting cell suspension was filtered through a sterile 70 µm nylon mesh filter (BD Falcon, Franklin Lakes, NJ, USA) to eliminate undigested tissue fragments. The filtrate was centrifuged at 200× *g* for 10 min at room temperature, after which the supernatant was discarded. The resulting cell pellet was resuspended in complete DMEM/F12 medium supplemented with 10% foetal bovine serum (FBS; Sigma-Aldrich, Cat. No. F7524, St. Louis, MO, USA) and 1% antibiotic-antimycotic solution (Sigma-Aldrich, Cat. No. A5955, St. Louis, MO, USA). Cell density and viability were assessed using a hemocytometer (Brand, Wertheim, Germany) with trypan blue (0.4%, Sigma-Aldrich, Cat. No. T8154, St. Louis, MO, USA) exclusion assay. For proliferation studies and hormone quantification assays, cells were seeded in *n* = 6 biological replicates per experimental group (derived from two independent cell isolation procedures, with 3 technical replicates each). For transcriptomic analysis, to obtain sufficient RNA concentrations, samples were pooled into 2–3 biological replicates per group from independent culture wells. For proliferation studies and hormone quantification assays, cells were seeded at a density of approximately 1–2 × 10^4^ cells/well in 24-well plates (NUNC, Roskilde, Denmark) or E-Plate 16 plates (Agilent Technologies, Santa Clara, CA, USA) for xCELLigence assays (see below). Cultures were incubated at 37 °C in a humidified atmosphere containing 5% CO_2_. Growth medium was replenished every 24 h unless specified otherwise.

### 2.2. Cell Viability Test and Experimental Design

The assessment of cell viability after ACTH treatment was conducted by means of the MTT (3-(4,5-dimethylthiazol-2-yl)-2,5-diphenyltetrazolium bromide) reduction assay, in accordance with the manufacturer’s instructions (Sigma-Aldrich, Cat. No. M2128, St. Louis, MO, USA) (Supplementary Figure S1). In summary, drug- or vehicle-treated cells were exposed to MTT solution at a final concentration of 0.5 mg/mL. Thereafter, the resulting formazan crystals were solubilised in DMSO (Sigma-Aldrich, Cat. No. D2650, St. Louis, MO, USA). Absorbance was subsequently measured at a wavelength of 570 nm, employing a spectrophotometer (Biotek, Winooski, VT, USA, Synergy 2).

Four experimental groups were established based on two variables: serum type and hormone treatment. Specifically, 24 h after initial seeding, half of the cultures were transitioned to DMEM/F12 medium containing charcoal-stripped FBS (designated as “S” for charcoal-stripped serum), while the remaining cultures were maintained in medium containing standard serum. In this study, charcoal-stripped serum refers specifically to the use of medium supplemented with charcoal-stripped foetal bovine serum, a standard method to reduce exogenous steroid content. Charcoal-stripped foetal bovine serum was obtained commercially (F6765, Sigma-Aldrich, St. Louis, MO, USA) and stored at −20 °C until use. This serum is prepared by the manufacturer through treatment with activated charcoal and dextran to reduce steroid hormones, growth factors, and other small molecules while preserving essential proteins. Importantly, charcoal particles are removed by filtration during the commercial preparation process, and no charcoal is present in the final serum product used for cell culture. The CSS-FBS was thawed and added to DMEM/F12 medium at a final concentration of 10% (*v*/*v*), identical to the concentration used for standard FBS, along with 1% antibiotic-antimycotic solution. We acknowledge that charcoal stripping is not specific for steroids and removes other serum components including growth factors and lipophilic molecules, which may contribute to the observed cellular responses. After an additional 24 h period, ACTH (1–24) (Sigma-Aldrich, Cat. No. A0298, St. Louis, MO, USA) was administered at a final concentration of 10 nM to the appropriate treatment groups. This concentration was chosen based on previous studies in rat adrenocortical cultures and adrenal cell lines showing robust steroidogenic responses without cytotoxicity (Supplementary Figure S1) [20,21]. Importantly, in our experiments no cytotoxic effects were observed at this concentration. Control groups received an equivalent volume of vehicle solution (PBS, Sigma-Aldrich, St. Louis, MO, USA or medium). Cultures were then incubated for a further 24 h period. Thus, the final experimental groups were designated as follows: Control, ACTH, Control(S), and ACTH(S).

### 2.3. Cell Proliferation Measurements

Real-time cell proliferation was assessed using the xCELLigence Real-Time Cell Analyzer (RTCA DP, Agilent Technologies, Santa Clara, CA, USA). Briefly, each well of the E-Plate 16 was initially filled with 50 µL of baseline medium, and a background impedance reading was recorded. Subsequently, adrenocortical cells were seeded at a density of 10,000 cells per well. Impedance values were recorded at 15 min intervals, which allowed the calculation of the Cell Index (CI). Normalised Cell Index (NCI) values were automatically generated by the RTCA software (version 1.2, Agilent Technologies, Santa Clara, CA, USA) by dividing the CI at each time point by the CI recorded at the onset of serum deprivation. Data were exported and plotted using R software (version 4.5.1, R Foundation for Statistical Computing, Vienna, Austria) with ggplot2 (version 4.0.0) and ggprism (version 1.0.7) packages to generate proliferation curves presented as mean ± standard deviation (SD).

### 2.4. Corticosterone Assays

Cell culture supernatants were collected and stored at −80 °C until corticosterone measurement. Corticosterone concentrations were quantified using a commercially available ELISA kit (Enzo Life Sciences, Cat. No. ADI-900-097, Farmingdale, NY, USA) following the manufacturer’s instructions. A standard curve ranging from 1 to 2500 ng/mL was established by plotting optical density (OD) against the log-transformed concentration values. Absorbance readings were performed at 450 nm with a reference wavelength of 450 nm using a Synergy 2 microplate reader (BioTek Instruments, Winooski, VT, USA). Sample concentrations were determined by interpolating the OD values onto the standard curve utilising a four-parameter logistic regression model. All samples were analysed in duplicate, and results (expressed as ng/mL) were statistically evaluated using the Kruskal–Wallis test followed by Dunn’s post hoc analysis. Statistically significant differences among groups were indicated by distinct letters on box-and-whisker plots.

### 2.5. RNA Extraction and Quality Control

To obtain higher concentrations of mRNA, pellets from cell cultures were pooled into two or three samples from which RNA was isolated. Total RNA was isolated directly from cell pooled pellets using a column-based purification method (RNeasy Mini Kit, Qiagen, Cat. No. 74104, Hilden, Germany). Briefly, cells were lysed in a guanidinium-thiocyanate-containing buffer, and the lysates were subsequently applied to spin columns. Following the recommended wash steps, purified RNA was eluted in nuclease-free water (Qiagen, Hilden, Germany). RNA concentration and purity were quantified using a NanoDrop spectrophotometer (Thermo Fisher Scientific, Waltham, MA, USA), confirming acceptable purity with A260/A280 ratios greater than 1.8. RNA integrity was assessed by capillary electrophoresis using an Agilent Bioanalyzer (Agilent Technologies, Santa Clara, CA, USA).

### 2.6. Microarray Analysis

Detailed procedures for microarray analysis have been previously described [22,23]. Briefly, total RNA (50–200 ng) from each experimental group was reverse-transcribed, amplified, and labelled according to the manufacturer’s WT Plus Kit protocol (Affymetrix, Santa Clara, CA, USA). The labelled cDNA was hybridised overnight at 45 °C onto Affymetrix GeneChip Rat Gene 2.1 ST Arrays (Affymetrix, Santa Clara, CA, USA). Post-hybridization processing, including washing and staining, was performed using an Affymetrix Fluidics Station (Affymetrix, Santa Clara, CA, USA). Arrays were scanned on an Affymetrix GeneChip Scanner (Affymetrix, Santa Clara, CA, USA), and initial quality control parameters (hybridization efficiency and labelling integrity) were evaluated using Affymetrix Expression Console software (version 2.0, Affymetrix, Santa Clara, CA, USA).

### 2.7. Bioinformatic and Statistical Analyses

All microarray data analyses were performed in R (version 4.5.1, R Foundation for Statistical Computing, Vienna, Austria) using Bioconductor packages. Raw CEL files were imported into R and processed using the robust multiarray average (RMA) algorithm within the oligo package (version 1.72.0), including background correction, normalisation, and summarization [24]. Expression values were log_2_-transformed. Probes exhibiting low variability were removed using the genefilter package (version 1.90.0). Differential expression analysis was conducted with limma (version 3.64.3) [25], applying pairwise contrasts: “ACTH vs. Control,” “ACTH(S) vs. Control(S),” and “Control vs. Control(S).” Differentially expressed genes (DEGs) were defined by a Benjamini–Hochberg false discovery rate (FDR)-adjusted *p*-value < 0.05 and absolute fold change ≥ 1.8. Significant DEGs were visualised via volcano plots (ggplot2) and heatmaps displaying the top 10 DEGs per contrast (ComplexHeatmap, version 2.24.1). Principal component analysis (PCA) was performed using the factoextra package (version 1.0.7) to visualise sample grouping [26,27].

### 2.8. Functional and Pathway Enrichment

Genes identified as significantly differentially expressed (FDR < 0.05 and fold change ≥ 1.8) underwent functional enrichment analysis using Gene Ontology (GO) via DAVID Bioinformatics Resources 6.8 (National Institute of Allergy and Infectious Diseases, NIH, Bethesda, MD, USA) [28], examining the Biological Process (BP), Molecular Function (MF), and Cellular Component (CC) categories. Enriched GO terms were considered significant at a Benjamini–Hochberg adjusted *p*-value < 0.05. Bubble plots were generated to illustrate the top ten significantly enriched and depleted GO terms, and detailed results were further visualised using heatmaps.

### 2.9. Gene Set Enrichment Analysis (GSEA)

Pre-ranked GSEA was conducted using the fgsea package (version 132.4) in R [29]. Genes were ranked by a metric combining log_2_ fold change and –log_10_(*p*-value) for each comparison. GSEA utilised curated gene sets from the Molecular Signatures Database (MSigDB) or GO Biological Processes, performing 10,000 permutations. Gene sets exhibiting normalised enrichment scores (NES) > ±1.5 and FDR < 0.05 were considered significantly enriched.

### 2.10. PathfindR Analysis

Additionally, PathfindR software (version 2.6.0, R package) was employed to identify significantly enriched KEGG or Reactome pathways using DEG log_2_ fold changes and adjusted *p*-values as input [30]. Pathways with an FDR < 0.05 were considered significant, with visual outputs including cluster heatmaps highlighting DEGs within these pathways.

### 2.11. Statistical Analysis

Non-microarray data (e.g., hormone assays, cell proliferation measurements) are presented as means ± standard deviation (SD) unless otherwise specified. Statistical comparisons among more than two groups were performed using the Kruskal–Wallis or one-way ANOVA tests, followed by appropriate post hoc analyses (Dunn’s or Tukey’s test). Adjusted *p*-values < 0.05 were considered statistically significant. Data visualisation and analyses were conducted in R using packages including ggplot2 (version 4.0.0), dplyr (version 1.1.4), and ComplexHeatmap (version 2.24.1).

## 3. Results

### 3.1. Effects of ACTH on Proliferation and Corticosterone Secretion Under Standard and Steroid-Depleted Conditions

**Figure 1:**
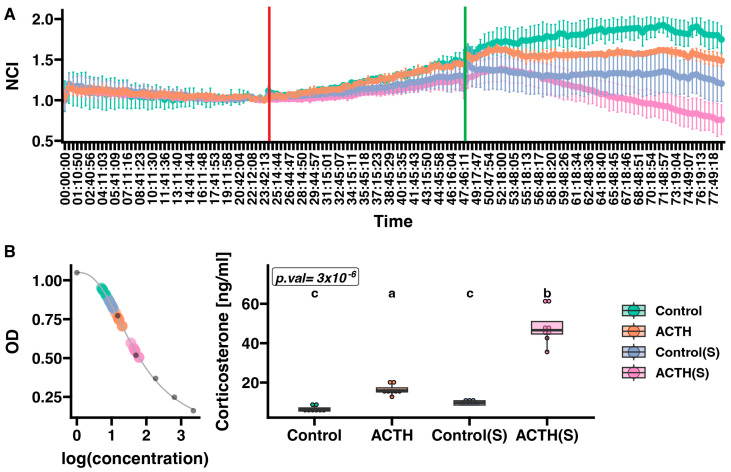
Effect of ACTH on proliferation (**A**) and corticosterone secretion (**B**) in primary cultures of rat adrenal cells. (**A**) Cell proliferation was continuously measured at 15 min intervals using the xCELLigence Real-Time Cell Analyzer (RTCA). The red line indicates the start of cell starvation, at which point charcoal-stripped serum medium (charcoal-stripped medium) was added to groups labelled “S”. The green line indicates the time at which ACTH was administered to the respective experimental groups. The RTCA chart presents mean normalised cell index (NCI) ± SD. (**B**) Corticosterone standard curve (left panel) with superimposed OD values for individual samples. The box plot (right panel) illustrates corticosterone levels across experimental groups. The Kruskal–Wallis test *p*-value is shown in the upper left corner of the plot. Results of Dunn’s post hoc test are indicated by different letters, with each letter representing statistically distinct groups. A uniform colour scale was applied to all charts, as presented in the legend on the right. Data represent mean ± SD from *n* = 6 biological replicates per group. Each data point on the box plot (panel B) represents an individual biological replicate from independent culture wells.

Proliferation of primary rat adrenocortical cells was measured in real time using the xCELLigence RTCA system over approximately 78 h, with four groups differing by serum supplementation (standard vs. charcoal-stripped, indicated as “S” and by treatment with ACTH or vehicle (Figure 1A). Cells in the “S” groups had their medium switched to charcoal-stripped FBS 24 h after seeding, whereas other groups remained in standard serum. Subsequently, at the 48 h, one charcoal-stripped group (“ACTH(S)”) and one standard-serum group (“ACTH”) were treated with ACTH (10 nM). The NCI was recorded at 15 min intervals until approximately 78 h. Analysis of the NCI at the final measured time point (*n* = 6 per group) revealed significant differences among the groups. Under standard serum conditions, the Control group achieved an NCI of 1.7487 ± 0.1643. In contrast, switching to charcoal-stripped medium alone (Control(S)) decreased the NCI to 1.2048 ± 0.2216—an approximate 31% reduction relative to the Control group. ACTH treatment in standard serum (ACTH) resulted in an NCI of 1.4875 ± 0.0781, representing a decrease of roughly 15% compared to the Control. Most notably, under the charcoal-stripped condition, ACTH treatment (ACTH(S)) further reduced the NCI to 0.7593 ± 0.1818, corresponding to an approximate 37% decrease relative to the Control(S) group.

Corticosterone levels varied substantially among the four experimental conditions (Figure 1B). In the Control group, cells secreted a median corticosterone level of 6.20 ng/mL (IQR: 5.50–7.08 ng/mL). In contrast, cells maintained in charcoal-stripped medium without ACTH (Control(S)) exhibited a median level of 9.70 ng/mL (IQR: 8.54–10.74 ng/mL). ACTH treatment under standard serum conditions (ACTH) led to a marked increase in corticosterone output, with a median of 15.98 ng/mL (IQR: 15.21–17.28 ng/mL). Notably, the highest corticosterone concentrations were observed in cells treated with ACTH in charcoal-stripped medium (ACTH(S)), which reached a median level of 46.60 ng/mL (IQR: 44.68–51.00 ng/mL). Statistical analysis using the Kruskal–Wallis test revealed a highly significant overall difference among the groups (*p* = 3 × 10^−6^). Dunn’s post hoc comparisons indicated that the Control and Control(S) groups did not differ significantly, whereas corticosterone levels in the ACTH group were significantly higher than those in both control groups, and levels in the ACTH(S) condition were significantly elevated relative to all other groups. The ACTH group exhibited lower variability (SD = 0.78) compared to other groups, reflecting the robust and consistent steroidogenic response to ACTH under stable culture conditions with standard serum supplementation. In contrast, the ACTH(S) group showed greater variability (SD = 2.56), likely reflecting individual culture-to-culture differences in adaptation to serum starvation conditions.

### 3.2. Serum Deprivation Enhances the Transcriptomic Response to ACTH in Primary Culture of Rat Adrenocortical Cells

**Figure 2:**
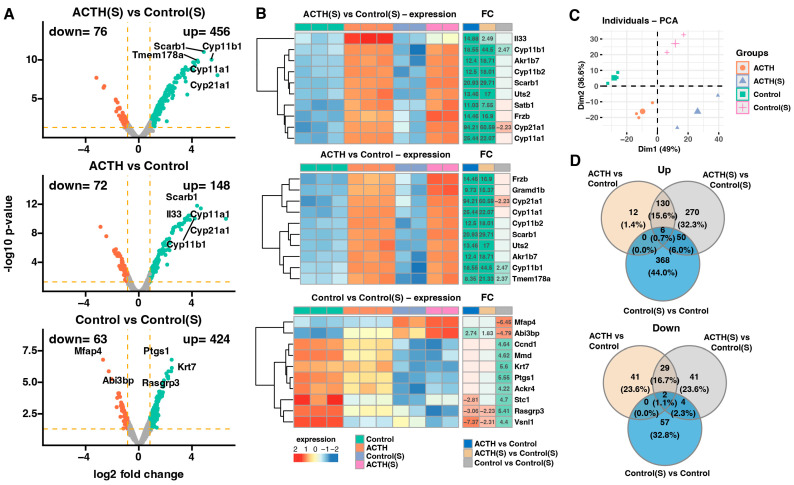
Transcriptomic analysis using microarrays. (**A**) Volcano plots illustrating differential gene expression for three pairwise comparisons: ACTH(S) vs. Control(S), ACTH vs. Control, and Control vs. Control(S). Genes meeting the significance threshold (adjusted *p*-value < 0.05 and fold change ≥ 1.8) are highlighted in red (downregulated) or turquoise (upregulated). The number of significantly downregulated and upregulated genes is indicated in the upper left and right corners of each plot, respectively. (**B**) Heatmaps showing the expression profiles of the 10 genes with the highest absolute fold-change values across comparisons. Each heatmap displays individual sample expression levels, gene symbols, and fold-change (FC). Numerical FC values are shown only for genes and comparisons that meet the predefined significance criteria (adjusted *p*-value < 0.05 and FC ≥ 1.8). (**C**) Principal component analysis (PCA) plot. Each point represents a single sample, colour-coded according to the experimental group. (**D**) Venn diagrams summarising the overlap of differentially expressed genes between comparisons. The upper diagram corresponds to significantly upregulated genes, while the lower diagram represents downregulated genes.

To investigate the transcriptional effects of ACTH stimulation under standard and steroid-depleted conditions, a comprehensive microarray-based gene expression analysis was performed. Differential expression analysis between the groups revealed robust transcriptomic changes, as visualised by volcano plots (Figure 2A). In the ACTH(S) vs. Control(S) comparison, a total of 532 genes were significantly differentially expressed (FDR < 0.05, |log2FC| ≥ 1.8), with 456 genes upregulated and 76 downregulated. Notably, key steroidogenic genes such as *Cyp11a1*, *Cyp11b1*, *Cyp21a1*, and *Scarb1* were among the top upregulated transcripts. Comparison of ACTH vs. Control cells revealed 220 differentially expressed genes (148 upregulated, 72 downregulated), also highlighting *Cyp11a1*, *Cyp11b1*, and *Cyp21a1* as consistently responsive to ACTH stimulation under standard conditions. In contrast, when comparing Control vs. Control(S), 487 transcripts were altered (424 upregulated, 63 downregulated), indicating that charcoal-stripped serum alone exerts a strong effect on gene expression.

The top 10 genes with the highest absolute fold changes for each pairwise comparison are presented as heatmaps (Figure 2B). Most notably, ACTH(S) treatment induced stronger upregulation of steroid biosynthesis-related genes compared to ACTH under standard serum conditions. This enhanced transcriptional response in ACTH(S) cells included robust induction of key steroidogenic genes such as *Cyp11a1*, *Cyp11b1*, *Cyp21a1*, *Star*, and *Scarb1*, which are involved in cholesterol uptake, transport, and conversion to glucocorticoids. Conversely, ACTH treatment under standard serum conditions resulted in a more modest upregulation of these genes, suggesting that the absence of endogenous steroids potentiates the steroidogenic gene processes. Furthermore, ACTH(S)-treated cells demonstrated heightened expression of metabolic and transporter genes, including *Akr1b7* and *Uts2*, indicating a more extensive functional activation. It is noteworthy that charcoal-stripped serum alone (Control(S) vs. Control) altered the expression of several genes related to cell structure and stress response, including *Rasgrp3*, *Abi3bp*, and *Ptgs1*, indicating that hormone-free conditions modulate basal transcription independently of ACTH. Collectively, these results lend support to the hypothesis that ACTH elicits a context-dependent transcriptional profile, with steroid-depleted environments enabling a stronger and more coordinated activation of steroidogenic and metabolic pathways.

The PCA was conducted to assess global variance in gene expression across experimental conditions. As shown in Figure 2C, the first two principal components, Dim1 and Dim2, accounted for 49% and 26% of the total variance, respectively. This dimensionality reduction revealed clear clustering of biological replicates within each group and distinct separation between groups. The most pronounced divergence was observed along Dim1, which effectively discriminated ACTH(S) samples from all other conditions, reflecting the dominant influence of ACTH stimulation under charcoal-stripped serum conditions. Meanwhile, Dim2 contributed to the separation between ACTH and Control groups under standard serum, suggesting a subtler but distinct ACTH-driven transcriptomic shift when endogenous steroids are present. Together, these results indicate that charcoal-stripped serum amplifies the transcriptional response to ACTH, and that both hormonal treatment and serum conditions contribute independently and interactively to gene expression profiles in studied adrenocortical cells.

To further characterise the relationship between gene expression responses to ACTH under different hormonal environments, Venn diagrams were generated to visualise the overlap of differentially expressed genes (DEGs) between conditions (Figure 2D). Among the transcripts that were found to be upregulated, 270 genes were uniquely induced in ACTH(S) compared to Control(S), while only 130 genes were shared between ACTH(S) and ACTH (standard serum), and a small subset of 12 genes was specific to ACTH vs. Control. These results emphasise the enhanced and largely distinct transcriptional activation triggered by ACTH in steroid-depleted conditions, which cannot be fully reproduced under standard serum. Furthermore, a comparative analysis of downregulated genes revealed 41 transcripts that were suppressed in the ACTH vs. Control cohort, and 29 of these transcripts were also identified in the ACTH(S) vs. Control(S) comparison. Notably, the ACTH(S) and Control(S) groups exhibited minimal overlap with other groups. This limited intersection suggests that the repressive effects of ACTH on gene expression are influenced by steroid availability and reflect condition-specific regulatory programmes.

These findings suggest that ACTH stimulation under charcoal-stripped conditions elicits a broader response. While we interpret this as being at least partly due to reduced steroid feedback, it is also possible that other components removed during serum stripping (e.g., growth factors) contributed to the observed effects. The enhanced activation of steroidogenic and metabolic gene networks, along with the context-specific repression of proliferative and structural pathways, underscores the modulatory role of steroid availability in shaping ACTH responsiveness. The findings of this study demonstrate that ACTH functions as a pivotal regulator of adrenal gene expression, and that its transcriptional impact is significantly influenced by the prevailing hormonal environment.

**Figure 3:**
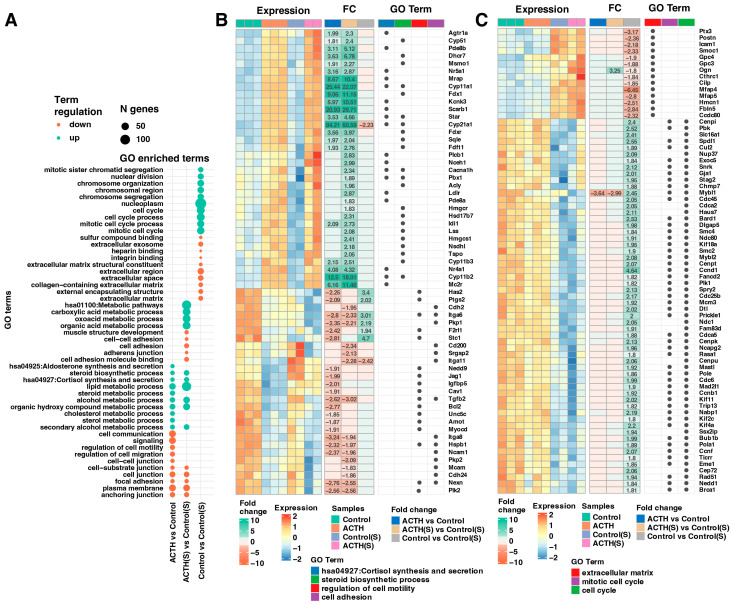
Functional Enrichment Analysis of Differentially Expressed Genes (DEGs). (**A**) Bubble plot illustrating the Gene Ontology (GO) enrichment analysis of DEGs. The top 10 upregulated and top 10 downregulated genes—ranked by adjusted *p*-value—were selected from three comparisons: ACTH(S) vs. Control(S), ACTH vs. Control, and Control vs. Control(S). DEGs were annotated and analysed using the DAVID database. (**B**,**C**) Combined heatmaps displaying the detailed expression profiles of DEGs associated with enriched GO terms. Panel (**B**) focuses on genes involved in the synthesis and secretion of cortisol, the steroid biosynthetic process, and the regulation of cell motility and adhesion. Panel (**C**) focuses on genes involved in the regulation of the cell cycle and the organisation of extracellular structures, including the extracellular matrix, the mitotic cell cycle and the cell cycle. In both panels, each row represents a gene and each column an individual sample. Gene symbols, expression profiles, and fold-change (FC) values are shown, with numerical FC values presented only for comparisons that meet the significance thresholds (adjusted *p*-value < 0.05 and FC ≥ 1.8). Additionally, dot annotations indicate the functional categorization of the genes.

To identify the biological processes underlying transcriptomic changes induced by ACTH stimulation and charcoal-stripped serum, we performed Gene Ontology (GO) enrichment analysis on differentially expressed genes (DEGs). As shown in Figure 3A, upregulated and downregulated genes were enriched in distinct sets of functional categories depending on the experimental comparison. In the ACTH(S) vs. Control(S) contrast, significantly upregulated genes were enriched in steroid hormone biosynthesis pathways, including steroid metabolic process, cholesterol biosynthetic process, and cortisol synthesis and secretion (e.g., *Cyp11a1*, *Cyp11b1*, *Star*, *Scarb1*) (Figure 3B). Moreover, this the ACTH(S) vs. Control(S) comparison shows also activation of GO terms such as lipid metabolism, organic acid metabolism, and mitochondrial function, highlighting the activation of a robust steroidogenic programme in response to ACTH under steroid-depleted conditions. Conversely, downregulated genes in the ACTH(S) group were found to be significantly enriched in pathways associated with cell adhesion, extracellular matrix (ECM) organisation, and cell–substrate junctions. This was particularly evident in the Control(S) vs. Control comparison, suggesting that charcoal-stripped serum alone suppresses proliferative and structural gene networks, which is further enhanced upon ACTH treatment (Figure 3C). In the ACTH vs. Control group, transcriptional changes were more modest. However, key steroidogenic genes were still significantly upregulated, albeit with lower fold changes compared to ACTH(S), indicating that the presence of serum-derived steroids may attenuate ACTH-induced transcriptional activation. Heatmaps of selected enriched GO terms (Figure 3B,C) illustrate representative gene expression profiles and fold changes across conditions. It is noteworthy that genes implicated in the mitotic cell cycle (e.g., *Cdk1*, *Cenpa*, *Mki67*) exhibited significant downregulation under charcoal-stripped serum conditions, particularly in Control(S) and ACTH(S), indicating that proliferative activity is repressed in favour of differentiation and steroidogenic output. Collectively, these findings suggest that ACTH stimulation in the absence of steroids promotes a coordinated upregulation of genes involved in steroidogenesis, while repressing cell cycle and adhesion-related genes, thereby shifting the cellular phenotype towards a more differentiated, hormone-producing state.

### 3.3. ACTH Reprograms Functional Pathways Depending on Steroid Availability

**Figure 4:**
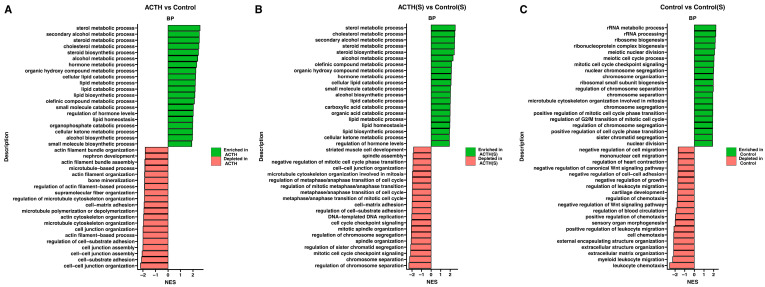
Gene Set Enrichment Analysis (GSEA) of Transcriptomic Data. Bar plots displaying the GSEA results for each pairwise comparison (**A**) ACTH vs. Control (**B**) ACTH(S) vs. Control(S), and (**C**) Control vs. Control(S). For each comparison, the top 10 gene sets with the most positive normalised enrichment scores (NES) and the top 10 with the most negative NES are shown. Bars are colour-coded to differentiate between gene sets enriched in the experimental group (positive NES) and those depleted (negative NES).

To further characterise the global biological programmes modulated by ACTH stimulation and serum deprivation, GSEA was performed using GO Biological Process (BP) terms. Normalised enrichment scores (NES) and directionality are summarised in Figure 4A–C. In the ACTH vs. Control comparison (Figure 4A), ACTH stimulation resulted in significant enrichment of steroid-related pathways, including steroid metabolic process, cholesterol biosynthetic process, secondary alcohol metabolism, and regulation of hormone levels. Conversely, negatively enriched gene sets comprised terms associated with cytoskeletal organisation, cell adhesion, and microtubule dynamics, suggesting a suppression of structural and proliferative functions. In ACTH(S) vs. Control(S) comparison (Figure 4B), the enrichment pattern was even more pronounced. Among the most positively enriched pathways were lipid biosynthetic process, steroid hormone biosynthesis, and cellular ketone metabolism, while terms related to cell junction organisation, mitotic division, and extracellular matrix structure were significantly downregulated. The present findings suggest that steroid starvation enhances the transcriptional sensitivity of cultured rat adrenocortical cells to ACTH, thereby promoting a shift toward steroidogenic specialisation and away from growth-associated programmes. The Control(S) vs. Control comparison (Figure 4C) further corroborated these observations: charcoal-stripped serum alone induced a significant downregulation of proliferative gene sets, including mitotic cell cycle, chromosome segregation, and microtubule organisation. The upregulation of gene sets in this comparison was predominantly associated with ribosomal biogenesis and rRNA metabolism, indicating a cellular stress response and reorganisation of fundamental metabolic activity in response to serum deprivation. Collectively, these data indicate that ACTH stimulation, particularly in the absence of exogenous steroids, drives a transcriptional reprogramming favouring steroid biosynthesis and metabolic activation, while concomitantly repressing proliferative and structural pathways typically associated with cell cycle progression and adhesion.

**Figure 5:**
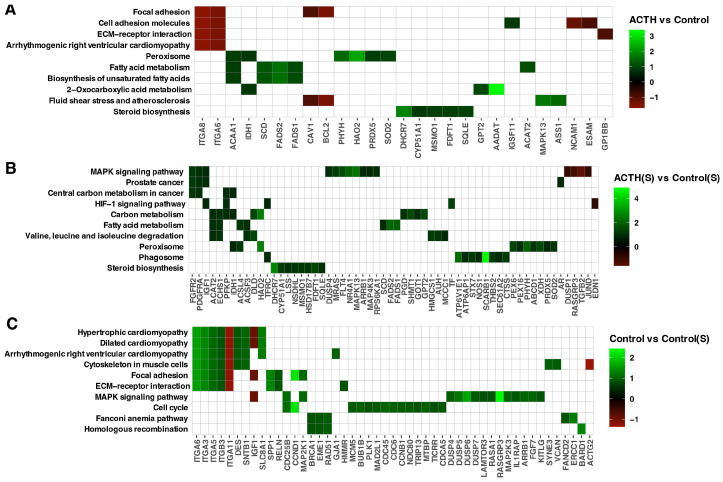
PathfindR Gene Enrichment Analysis. Enriched biological pathways were identified for three experimental comparisons: (**A**) ACTH vs. Control, (**B**) ACTH(S) vs. Control(S), and (**C**) Control vs. Control(S). The heatmap displays ten enriched pathways along the vertical axis, with the corresponding differentially expressed genes arranged horizontally. Colour-coded square annotations represent the log_2_ fold-change (log_2_FC) values.

Utilising the PathfindR tool, pathway enrichment analysis was conducted to contextualise the biological relevance of ACTH and serum deprivation-induced gene expression changes. This facilitated the visualisation of differentially expressed genes (DEGs) mapped onto significantly enriched KEGG pathways for each pair-wise comparison (Figure 5A–C). In the ACTH vs. Control comparison (Figure 5A), the enriched pathways included steroid biosynthesis, fatty acid metabolism, peroxisome, and biosynthesis of unsaturated fatty acids. Genes that were found to be upregulated included *CYP51A1*, *FDFT1*, *SQLE* and *DHCR7*, which have been identified as central components of the steroidogenic axis. In contrast, genes associated with focal adhesion and ECM–receptor interaction (e.g., *ITGA6*, *ITGA8*, *BCL2*) were found to be downregulated, suggesting a suppression of structural and proliferative features. In the ACTH(S) vs. Control(S) comparison (Figure 5B), pathway-level changes were broader and more pronounced. Enrichment was observed for MAPK signalling, carbon metabolism, HIF-1 signalling, and valine/leucine degradation pathways. It is noteworthy that genes implicated in steroid biosynthesis (*HSD17B7*) and lipid metabolism were found to be significantly activated under conditions of steroid depletion. This further corroborates the notion of enhanced ACTH responsiveness. Furthermore, the analysis identified the enrichment of several oncogenic and metabolic pathways, including central carbon metabolism in cancer and prostate cancer. This finding suggests the potential for cellular remodelling in response to nutrient limitations. For the Control(S) vs. Control comparison (Figure 5C), serum deprivation alone altered pathways linked to cell cycle, cytoskeletal regulation, and DNA repair, with reduced expression of BUB1. These findings suggest that the removal of endogenous steroids suppresses cell cycle progression and structural maintenance, independently of ACTH. Overall, pathway-based mapping supports the conclusion that charcoal-stripped serum enhances the steroidogenic programme induced by ACTH while dampening proliferative and structural gene networks, and that these effects are mediated through distinct metabolic, hormonal, and signalling routes.

## 4. Discussion

In this study, we demonstrate that the response of primary rat adrenocortical cells to ACTH is significantly modulated by the presence or absence of an extracellular steroid in culture medium. Moreover, we indicate that ACTH [10nM] do not have an impact on rat primary adrenocortical cell viability. The absence of a quantifiable decline in viability following 24 h of exposure to 10nM ACTH is consistent with the extensive literature that characterises ACTH as a trophic rather than a cytotoxic stimulus for adrenal cells. In vitro assays utilising guinea pig adrenal explants have demonstrated that synthetic ACTH 1-24 promotes growth at concentrations far below the nanomolar range (10–25 fg/mL^−1^), producing biphasic dose–response curves without any indication of cell death [31]. In addition, both in vivo and ex vivo studies conducted on rats demonstrated that pharmacological doses of ACTH resulted in the stimulation of capsular fibroblast proliferation and an increase in adrenal weight. However, these doses did not induce any overt toxicity within the cortical parenchyma [32]. In the next study, ACTH was administered at both physiological and supra-physiological doses to mice bearing MC2-R-positive adrenocortical tumours [33]. The primary effect of this administration was a dose-dependent reduction in tumour proliferation, without any alterations in apoptosis or overall organ weight. These results suggest that ACTH exerts a selective anti-proliferative action that does not affect normal cell survival [33]. The biphasic modulation of lymphocyte DNA synthesis by ACTH serves to illustrate that the hormone can enhance cellular activity at low concentrations while inhibiting it at higher levels. However, neither of these scenarios is associated with cellular toxicity [34]. The findings, taken collectively, lend further support to the observation that 10 nM ACTH does not compromise the viability of primary rat adrenal cortical cells, and it is consistent with our previous studies [20,21]. This observation serves to reinforce the role of ACTH as a growth-modulating factor rather than a cytotoxic agent.

In line with the formulated hypothesis, removing endogenous steroids using charcoal-stripped serum markedly enhanced the transcriptional and functional response to ACTH. While these results are consistent with previous observation, in the current study we have revealed the molecular basis of this phenomenon. This was demonstrated by increased corticosterone secretion and substantial upregulation of key steroidogenic genes, including *Cyp11a1*, *Cyp11b1*, *Star* and *Scarb1*. This increased hormonal output was accompanied by substantial suppression of cell proliferation, as confirmed by real-time impedance-based assays and downregulation of proliferation-related genes, such as *Mki67*, *Cdk1*, and *Bub1*. Moreover, these results confirm that the responsiveness of adrenocortical cells to ACTH is not fixed, but rather depends strongly on the hormonal context, particularly on the availability of negative feedback signals. Further analysis using functional enrichment methods (GO, GSEA and PathfindR) showed that, in primary cultures of rat adrenocortical cells, ACTH stimulation without steroids selectively activates pathways linked to steroid biosynthesis and lipid metabolism. At the same time, it suppresses pathways involved in the cell cycle, cytoskeletal organisation and extracellular matrix remodelling. This functional reprogramming suggests a shift in cellular priorities towards hormone production and away from programmes associated with proliferation or structural maintenance [35,36]. The PCA revealed clear segregation between the experimental groups, demonstrating strong intra-group consistency and thus underscoring the robustness of the transcriptomic data. This analysis confirmed the distinct gene expression profile of ACTH-treated, steroid-deprived cells. Venn diagram analysis also indicated that most ACTH-responsive genes in this condition were uniquely regulated.

The transcriptional effects observed in response to ACTH, particularly under steroid-depleted conditions, are classically attributed to the activation of the MC2R–cAMP–PKA signalling axis [37,38,39]. This pathway has been shown to increase the expression of steroidogenic genes by phosphorylating important transcription factors such as *CREB* and *SF-1*. This leads to the activation of StAR, CYP11A1 and other enzymes [7,40]. However, the extensive transcriptomic remodelling observed in ACTH(S)-treated cells, in terms of both gene number and functional enrichment, suggests that additional signalling routes may be involved. Specifically, analysis of gene sets related to MAPK, HIF-1 and lipid regulatory pathways, as revealed through GSEA and PathfindR, indicates potential crosstalk between ACTH-induced steroidogenesis and stress-responsive metabolic signalling. Previous studies have shown that prolonged exposure to ACTH or increased cAMP levels can activate the ERK1/2 and PI3K/AKT pathways, thereby contributing to context-specific gene regulation and cellular adaptation [7,40,41]. Therefore, our data are consistent with the view that in vitro ACTH acts via both canonical and non-canonical signalling mechanisms. At the same time, the downregulation of proliferation-associated genes and the upregulation of steroidogenic genes observed are consistent with the established ACTH–MC2R–cAMP–PKA pathway. This reduces the likelihood that these effects were caused by non-specific or off-target signalling.

The observed transition from proliferation to steroidogenic activation in ACTH-treated, charcoal-stripped serum cells may be a way in which the cells adapt to endocrine stress. In physiological contexts such as chronic ACTH exposure during adrenal insufficiency or prolonged stress, adrenocortical cells prioritise steroid hormone synthesis over cellular replication to maintain systemic homeostasis. A similar trade-off occurs in developmental contexts, such as in the foetal adrenal cortex, where cells gradually transition from a proliferative state to a differentiated steroidogenic state [42,43]. Our findings suggest that this in vitro model may recapitulate this conserved biological strategy, which is aimed at restoring glucocorticoid levels when systemic steroid feedback is absent. In this regard, charcoal-stripped serum in vitro can be considered an indicator of reduced feedback inhibition. This results in increased sensitivity to ACTH and steroidogenesis. Potential mechanisms that have been identified include increased *MC2R* expression and decreased glucocorticoid-mediated repression. Although these parallels offer valuable insights, direct extrapolation to humans necessitates validation in primary human adrenal cultures or validated cell lines.

Our results also demonstrate a high level of agreement with previous studies on adrenal cell lines. For example, research using the human H295RA adrenocortical cell line has shown that ACTH can significantly increase the production of aldosterone, cortisol and dehydroepiandrosterone, as well as upregulating the expression of the MC2R receptor and multiple steroidogenic enzymes [44]. This mirrors the upregulation pattern observed in our model, suggesting conservation of ACTH-induced transcriptional programmes across species. Similarly, ACTH has been shown to exert a biphasic effect on the cell cycle in Y-1 mouse adrenocortical cells: short-term stimulation promotes S-phase entry, while prolonged exposure inhibits DNA synthesis [45]. Consistent with this, we found that 24 h treatment with ACTH under steroid-depleted conditions significantly reduced cell proliferation. Unlike immortalised cell lines, however, our use of primary cells enables the detection of more physiologically relevant, context-dependent responses. This includes alterations in gene expression associated with the extracellular matrix (ECM), which fulfils a pivotal regulatory function in determining adrenocortical cell fate. In the present study, the administration of ACTH in the absence of steroids resulted in the repression of numerous ECM- and adhesion-related genes, including *Cdh1*, *Itga6*, and *Timp3*. As has been previously reported, ECM components in the adrenal cortex exhibit zonal specificity. For instance, fibronectin and laminin are differentially expressed along the corticomedullary axis and are associated with specific cellular functions [46,47]. It is important to note that the Gallo-Payet group demonstrated that fibronectin and collagens I/IV enhance steroidogenesis, whereas laminin supports proliferation [48]. Our findings of this study suggest that ACTH-driven suppression of ECM-related gene expression may contribute to a shift in the microenvironment that favours steroidogenic activation over cell proliferation. This additional regulatory layer may be of relevance in primary tissue, where ACTH responses are known to involve not only intracellular signalling but also interaction with the structural and mechanical properties of the local niche.

In the light of our research and the suggestions reached, the question arises whether similar mechanisms of ACTH action are observed in vivo. In her very interesting monograph, Dallman analyses the effects of corticotropin on the adrenal glands [49]. She finds that ACTH first induces hypertrophy, i.e., an increase in the volume of cells in the rat adrenal cortex, followed by stimulation of hyperplasia, i.e., an increase in their number. This suggests that both in vitro and in vivo the mechanism of action of ACTH on secretory activity and proliferation of adrenal cortex cells is very similar. Analogous conclusions can also be drawn from studies by other authors [17].

Nevertheless, not all results fully aligned with the literature. For instance, Janes et al. [50] reported that prolonged ACTH stimulation in Y-1 cells promoted proliferative gene expression via MAPK/ERK signalling, whereas in our study, particularly under ‘S’ conditions, we observed the opposite effect [51,52]. These discrepancies may be due to differences in cell type (transformed versus primary), ACTH dosage or stimulation duration. Additionally, the downregulation of proliferation-related genes and the suppression of cell cycle pathways observed here support previous findings indicating a functional trade-off between hormone production and proliferation in steroidogenic tissues [36,53]. The enrichment of cholesterol metabolism-related genes, particularly those involved in cholesterol transport and mitochondrial import, further corroborates this shift towards terminal differentiation and increased steroid output [54].

We are aware that our research may have some limitations. This study was conducted in vitro using pooled primary rat adrenocortical cells, which, although physiologically relevant, lack the complexity of the in vivo adrenal environment, including zonation, systemic hormonal inputs, and immune or vascular signals. The use of charcoal-stripped serum may have introduced non-specific metabolic stress beyond steroid depletion. We acknowledge that charcoal-stripped serum not only reduces exogenous steroid levels but may also remove other serum components, such as growth factors or lipids, which could contribute to reduced proliferation and altered gene expression. Thus, the observed effects are likely multifactorial and not solely attributable to steroid depletion. Furthermore, transcriptomic analyses were limited to a single ACTH dose and time point, without protein-level validation. While these factors constrain the resolution of zonal and systemic effects, the model robustly captured key ACTH-induced shifts in steroidogenic and proliferative programmes. Steroid levels in FBS and CSS were not assessed in this study. Considering that corticosterone secreted by adrenal cells rapidly exceeds serum-derived concentrations, we interpret the observed effects as likely influenced by both reduced exogenous steroids and changes in other serum components. Future studies including direct serum profiling will help to disentangle these factors. Although our study was performed in rat primary cells, the findings may have translational relevance. Enhanced ACTH responsiveness under conditions of reduced steroid feedback could mimic physiological or pathological states. Nevertheless, direct extrapolation to humans is limited, and future studies using human adrenal cell lines or primary human cultures will be required to validate these mechanisms. While our study provides robust functional and phenotypic validation of the transcriptomic findings, we acknowledge that targeted qPCR confirmation of individual gene expression changes could provide additional technical validation. However, the direct functional validation through corticosterone measurements and real-time proliferation assays, performed on the same biological samples used for transcriptomic analysis, demonstrates that the observed gene expression changes translate into measurable biological outcomes. This functional validation approach, combined with pathway-level bioinformatic confirmation and concordance with published literature, provides strong evidence for the reliability of our transcriptomic findings. Future studies could complement these findings with targeted gene-level validation to further refine our understanding of individual regulatory nodes within the identified pathways. Moreover, future work should include time-course studies, protein validation, and spatially resolved transcriptomic analyses to refine these observations.

To conclude, our findings demonstrate that in vitro extracellular steroid availability plays a critical role in shaping the transcriptional and functional response of adrenocortical cells to ACTH. Charcoal-stripped serum not only amplified the steroidogenic effects of ACTH but also reprogrammed the cellular phenotype toward differentiation at the expense of proliferation. These results highlight the dynamic interplay between endocrine signals and the local hormonal environment in regulating adrenal function. Beyond improving our understanding of adrenal cell plasticity, this work provides a valuable in vitro model for exploring the molecular mechanisms of steroidogenesis and ACTH sensitivity. As such, our data may inform both experimental endocrinology and the development of diagnostic or therapeutic strategies targeting adrenal dysfunction. Further research will be essential to validate these findings in vivo and to dissect zone-specific regulatory mechanisms within the adrenal cortex.

## Data Availability

The raw and processed microarray data supporting the conclusions of this article have been submitted to the ArrayExpress under the accession number E-MTAB-16213. Custom R analysis scripts are available from the corresponding author upon reasonable request.

## Supplementary Materials

The following supporting information can be downloaded at: https://www.mdpi.com/article/10.3390/cells14231844/s1. Figure S1: Effect of ACTH (10 nM) on the viability of primary rat adrenocortical cells after 24 h.
