# Supplementary material for: Serum Starvation Affects the Transcriptomic and Proliferative Response to ACTH in Primary Cultures of Rat Adrenocortical Cells
Source: Cells. 2025 Nov 22;14(23):1844. doi: 10.3390/cells14231844 (PMC12691138; doi:10.3390/cells14231844)
Supplement: Supplementary file 1 [file cells-14-01844-s001.zip › cells-3967610-supplementary.pdf]

Supplementary Figure S1

Effects of ACTH on cell viability

To assess the potential cytotoxic effect of ACTH on adrenocortical cells, cell viability was measured after 24 h of treatment with 10 nM ACTH in primary rat adrenocortical cultures using the MTT assay. As demonstrated in Figure 1, ACTH exposure did not result in a significant alteration in cell viability when compared with the untreated control group (98.5 ± 2.1% vs. 100 ± 2.6%, respectively). These findings demonstrate that ACTH at the tested concentration was not cytotoxic and did not impair cell survival under the experimental conditions.

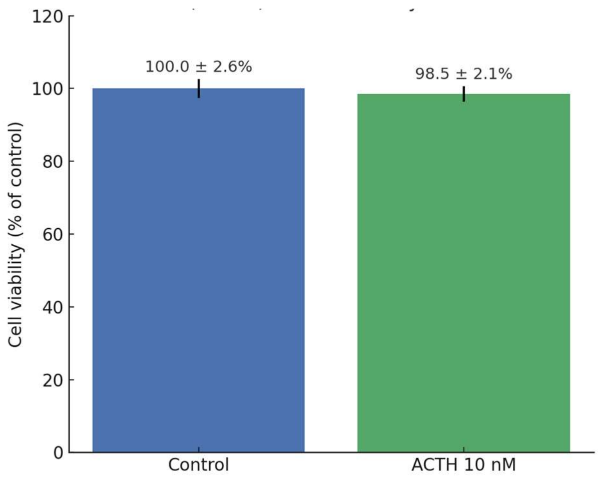

Figure S1. Effect of ACTH (10 nM) on the viability of primary rat adrenocortical cells after 24 h. Cell viability was determined using the MTT assay and expressed as percentage of control (mean ± SEM). ACTH treatment did not significantly affect cell viability, indicating the absence of cytotoxic effects under the tested conditions.
